# Supplementary material for: Disruption of Poly(ADP-ribosyl)ation Improves Plant Tolerance to Methyl Viologen-Mediated Oxidative Stress via Induction of ROS Scavenging Enzymes
Source: Int J Mol Sci. 2024 Aug 29;25(17):9367. doi: 10.3390/ijms25179367 (PMC11395660; doi:10.3390/ijms25179367)
Supplement: Supplementary file 1 [file ijms-25-09367-s001.zip › Table S1 Supplementary.pdf]

Table S1. Primers used for quantitative RT-PCR

Primer	5'-3' sequence	Reference	Primer concentration (nM)	E (%)
NbUBI3-F<br>NbUBI3-R	AATGTGAAAGCCAAGATCCAAG<br>CGGAGGCGGAGCACGAGATGAA	GenBank<br>TC20187	300	91.2
NbL23-F<br>NbL23-R	AAGGATGCCGTGAAGAAGATGT<br>GCATCGTAGTCAGGAGTCAACC	GenBank<br>TC19271	350	97
NbPARP1-F<br>NbPARP1-R	AACTGGAGGCACAGACTAAAGCCT<br>ACTGAGATAGCCCATTAGCAGCCTG	GenBank<br>KP771975	340	97
NbCAT2-F<br>NbCAT2-R	CGGAGGAGCAAATCACAG<br>CCACAATAGAAGGGCAGA	Zhu et al., 2020	350	96
NbMnSOD-F<br>NbMnSOD-R	AGACCTTTTCGCTCCCCG<br>CCACACCAAGCCACACCC	Zhu et al., 2020	340	97
NbAPX5-F<br>NbAPX5-R	GAGAGCATTTAGGAGAGG<br>TCAGCAGACAAGGACCAG	Zhu et al., 2020	330	95
NbGR-F<br>NbGR-R	AAGACAAGCAAAGTTCTGGG<br>CACAGTGGCATCAAAGTCC	Zhang et al. 2023	320	92

Zhu F, Zhu P-X, Xu F, Che Y-P, Ma Y-M,Ji Z-L. Alpha-momorcharin enhances Nicotiana benthamianaresistance to tobacco mosaic virus infection throughmodulation of reactive oxygen species. Molecular PlantPathology. 2020; 21:1212–1226. https://doi.org/10.1111/mpp.12974

Zhang D, Gao Z, Zhang H, Yang Y, Yang X, Zhao X, Guo H, Nagalakshmi U, Li D, Dinesh-Kumar SP, Zhang Y. The MAPK-Alfin-like 7 module negatively regulates ROS scavenging genes to promote NLR-mediated immunity. Proc Natl Acad Sci U S A. 2023 Jan 17;120(3):e2214750120.
